# GABAergic Neuron Deficit As An Idiopathic Generalized Epilepsy Mechanism: The Role Of BRD2 Haploinsufficiency In Juvenile Myoclonic Epilepsy

**DOI:** 10.1371/journal.pone.0023656

**Published:** 2011-08-24

**Authors:** Libor Velíšek, Enyuan Shang, Jana Velíšková, Tamar Chachua, Stephania Macchiarulo, Giorgi Maglakelidze, Debra J. Wolgemuth, David A. Greenberg

**Affiliations:** 1 Department of Cell Biology & Anatomy, New York College of Medicine, Valhalla, New York, United States of America; 2 Department of Pediatrics, New York College of Medicine, Valhalla, New York, United States of America; 3 Department of Obstetrics and Gynecology, New York College of Medicine, Valhalla, New York, United States of America; 4 Dominick P. Purpura Department of Neuroscience, Albert Einstein College of Medicine, Bronx, New York, United States of America; 5 Division of Statistical Genetics, Department of Biostatistics, Mailman School of Public Health, Columbia University Medical Center, New York, New York, United States of America; 6 Department of Psychiatry, Columbia University Medical Center, New York, New York, United States of America; 7 Department of Genetics and Development and Obstetrics and Gynecology, The Herbert Irving Comprehensive Cancer Center, and The Institute of Human Nutrition, Columbia University Medical Center, New York, New York, United States of America; 8 The Saul R. Korey Department of Neurology, Albert Einstein College of Medicine, Bronx, New York, United States of America; 9 Dominick P. Purpura Department of Neuroscience, Albert Einstein College of Medicine, Bronx, New York, United States of America; Consejo Superior de Investigaciones Cientificas, Spain

## Abstract

Idiopathic generalized epilepsy (IGE) syndromes represent about 30% of all epilepsies. They have strong, but elusive, genetic components and sex-specific seizure expression. Multiple linkage and population association studies have connected the bromodomain-containing gene *BRD2* to forms of IGE. In mice, a null mutation at the homologous *Brd2* locus results in embryonic lethality while heterozygous *Brd2+/−* mice are viable and overtly normal. However, using the flurothyl model, we now show, that compared to the *Brd2+/+* littermates, *Brd2+/−* males have a decreased clonic, and females a decreased tonic-clonic, seizure threshold. Additionally, long-term EEG/video recordings captured spontaneous seizures in three out of five recorded *Brd2+/−* female mice. Anatomical analysis of specific regions of the brain further revealed significant differences in *Brd2+/− vs +/+* mice. Specifically, there were decreases in the numbers of GABAergic (parvalbumin- or GAD67-immunopositive) neurons along the basal ganglia pathway, i.e., in the neocortex and striatum of *Brd2+/−* mice, compared to *Brd2+/+* mice. There were also fewer GABAergic neurons in the substantia nigra reticulata (SNR), yet there was a minor, possibly compensatory increase in the GABA producing enzyme GAD67 in these SNR cells. Further, GAD67 expression in the superior colliculus and ventral medial thalamic nucleus, the main SNR outputs, was significantly decreased in *Brd2+/−* mice, further supporting GABA downregulation. Our data show that the non-channel-encoding, developmentally critical *Brd2* gene is associated with *i)* sex-specific increases in seizure susceptibility, *ii)* the development of spontaneous seizures, and *iii)* seizure-related anatomical changes in the GABA system, supporting *BRD2*'s involvement in human IGE.

## Introduction

Idiopathic generalized epilepsy (IGE) is mostly genetic in origin [1] and represents about 30% of all epilepsies [2]. IGE comprises several sub-syndromes including Juvenile Myoclonic Epilepsy (JME) and Juvenile Absence Epilepsy (JAE) [3]. Although some channel genes have been shown to cause some rare forms of epilepsy [4], none have yet been proven to play a major role in the far more common IGEs. Few IGE genes have been identified, and those mostly through linkage scans chosen through specific phenotypes. Of those genes only *BRD2*, which is not a channel gene, has been both linked and associated in multiple studies with a particular IGE, JME [5], [6], [7], [8], [9], [10], [11], [12]. The statistical and population evidence supports *BRD2* as strongly influencing susceptibility to JME in particular and, potentially, a wider range of IGE syndromes/seizures, including photosensitivity [13], [14] and epilepsy-related electroencephalography (EEG) traits [6], [8].

### Statistical evidence supporting *BRD2* as the EJM1 locus for JME

Several linkage and association studies support *BRD2* as the EJM1 locus. The 6p21 locus was the first identified locus for a common epilepsy (JME) [6], a finding independently replicated [7] and confirmed again by Sander et al. [15], and in an independent data set by Greenberg et al. [10]. Durner et al. [8] demonstrated that the same locus led to the generalized electroencephalogram (EEG) abnormality seen in both JME cases and in family members unaffected with epilepsy and Tauer et al.[14] found linkage of 6p21 to the phenotype of photosensitivity using EEG. Greenberg et al. [10] further found evidence of an association to a microsatellite marker in the *BRD2* gene, subsequently substantiated by Pal et al. [11]. Later, in a follow-up to the Tauer et al. findings, Lorenz et al. [13] showed association of *BRD2* alleles to photosensitivity.

Cavalleri et al. [12] examined 5 different populations and confirmed the association of JME to *BRD2* in two of those populations: British and Irish. Two other populations, Indian and Australian, did not show association. This locus had previously been shown only Caucasian populations [10], [15], which was substantiated when no association was found in the Indian population. The ethnic makeup of the Australian population was unknown. The fifth population, German, illustrates the problems in replication in association studies because this same population showed linkage and association of *BRD2* to the EEG trait (see above). Because the evidence supports a role for BRD2 in epilepsy-related brain function, finding the biological basis for its influence on seizure susceptibility and abnormal (epileptiform) EEG traits will help elucidate the mechanisms underlying the etiology of the IGEs.

One of the problems in drawing conclusions from association studies, especially when comparing two or more populations, is that the existence of multiple disease-related alleles can make data interpretation difficult. Two reports illustrate the confounding factors in association studies of JME and the related EEG traits.

In one report, Layouni et al. [16] found an association of JME with the *TAP1* gene in Tunisians. However, the authors found no *TAP1* association in Caucasians. That *BRD2* does not associate with JME in some non-Caucasian populations has previously been demonstrated [10], [12], [15], [17]. Furthermore, given the close proximity of *TAP1* to *BRD2*, one could speculate that, in Tunisians, a DNA variant in *TAP1* affects expression of *BRD2*. Such “long distance” interactions have been reported in the past (e.g., [18]). Moreover, a *Tap1* knockout mouse shows no effects on brain development [19], in contrast to the profound effects on neural development in *Brd2−/−* mouse embryos [20] and our observations on *Brd2+/−* mice in the present study.

In a second report, de Kovel et al. [21] used a Dutch sample of IGE patients to test for association of three *BRD2* SNPs with the IGE phenotype and, in a smaller sample, the JME phenotype. They found no evidence of association of the three SNPs with those phenotypes. However, the SNPs used by de Kovel et al. were those reported associated, not with IGE or JME, but with EEG photosensitivity in a study by Lorenz et al. [13]. Pal [11] had tested JME (but not IGE) and had included only two of the three SNPs tested by de Kovel et al. One of those SNPs showed no association with JME in the Pal et al. report and one showed marginal association (although other SNPs and SNP haplotypes showed strong association evidence). de Kovel et al. rightly conclude that the data neither confirm nor refute the BRD2 association evidence. While de Kovel et al. did not test association of these SNPs with photosensitivity in that work, in a later report, de Kovel et al. [22] found no association of selected SNPs in *BRD2* with a photosensitivity subtype using, among others, the SNPs identified in the Lorenz et al. photosensitivity study. However, the de Kovel et al. cases were a mixture of IGE subtypes, thus perhaps diluting any specific *BRD2*-related effect. Indeed, several studies show that the chromosome 6p21 locus does *not* predispose to non-JME IGEs [9], [23], [24]. Mixtures of patients with different phenotypes would be less likely to be revealing.

Thus, because of the problems in interpretation due to different populations, differing phenotypes, and the number of possible different disease-related variants, association studies can take one only so far in the attempt to identify the disease locus, the responsible allele(s), and a possible biological mechanism. Eventually, statistical evidence must point the way to seeking biological evidence. We now have molecular biological data suggesting an epilepsy-related mechanism related to *BRD2*. Although the data are preliminary, we have observed that the ratio of normally-spliced to alternatively spliced (and non-functional) *BRD2* RNA is a function of the number of tandem GT repeats in a microsatellite located in intron 2 of *BRD2* [25]. If the ratio is small enough, such a situation could mimic haploinusfficiency. Thus, it is possible that several different alleles (or GT repeat lenths) may predispose to the brain-related phenomena that we report here and that associations may differ greatly depending on the frequencies of those alleles in the population.

*BRD2* is a member of the ‘BET’ subfamily of genes carrying bromodomain motifs [26] that includes the genes (mouse/human designation) *Brd2/BRD2*, *Brd3/BRD3*, *Brd4/BRD4*, and *Brdt/BRDT.* The zebrafish homologue of the *Brd2/BRD2* gene is highly expressed in the egg, early embryo, and developing nervous system [27]. Various functions have been ascribed to the BRD2 protein, including transactivation of promoters of several cell cycle regulatory genes [28], binding to mitotic chromosomes[29], and interaction with acetylated lysine-12 in histones [30] but its role in basic cellular functions *in vivo* remains unknown.

We generated a null mutation of the murine *Brd2* gene using a gene-trap approach [20]. Heterozygous *Brd2+/−* mice are viable and overtly normal. In contrast, the *Brd2*-null mutation (*Brd2−/−*) is incompatible with life: *Brd2−/−* mice die by embryonic day 11 and exhibit abnormal brain structures [20]. These observations suggest that *Brd2* is essential for neural development, observations subsequently confirmed by others [31]. Heterozygous *Brd2+/−* mice are viable and overtly normal. Understanding how *Brd2* functions during neural development in the mouse model will give us insight into basic mechanisms of IGE, and JME in particular.

In the present study, we determined if the viable and overtly healthy heterozygous *Brd2+/−* mice have increased susceptibility to flurothyl-induced seizures and if they develop spontaneous seizures. We further examined whether alterations in seizure susceptibility are associated with changes in GABAergic markers in the basal ganglia pathway involving cortex, striatum, substantia nigra pars reticulata (SNR), superior colliculus (SC), and ventral medial thalamic nucleus (VM). These areas were chosen because we found that the *Brd2+/−* mice display increased susceptibility to primarily generalized seizures, and GABAergic mechanisms in the SNR and SC have significant roles in controlling those seizure types [32], [33], [34], [35], [36], [37]. Changes in GABAergic neuron number within an endogenous seizure-controlling network could account for, or contribute to, increased seizure sensitivity or development of spontaneous seizures. Indeed, we here demonstrate that the viable and overtly healthy heterozygous *Brd2+/−* mice [20] not only show increased seizure susceptibility but develop spontaneous generalized seizures with corresponding EEGs abnormalities. We further show that the likely mechanism for this susceptibility is a decrease in GABAergic neurons in the basal ganglia pathway, including in the SNR, a critical seizure-controlling site [33]. Our results suggest that a mechanism underlying JME is, in part, an impairment in GABAergic inhibition due to wide-spread, developmentally-determined, abnormally low numbers of inhibitory neurons.

## Results

### Seizure susceptibility

We first asked whether heterozygous *Brd2+/−* knockout mice show increased seizure susceptibility with the “threshold test” using flurothyl exposure by inhalation. Decreased thresholds for flurothyl-induced tonic-clonic seizures were observed in *Brd2+/− vs. Brd2+/+* female, but not male, mice, while *Brd2+/−* males showed decreased thresholds for clonic seizure induction.

**Figure 1:**
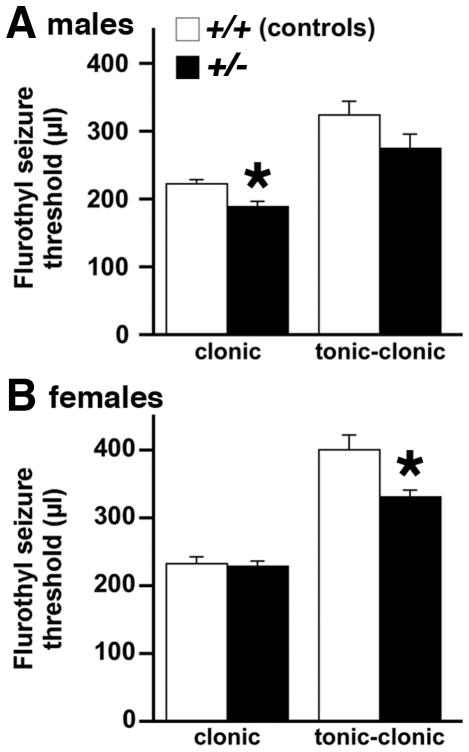
Susceptibility of *Brd2* heterozygous KO mice (*+/-*) and control littermates (+/+) to flurothyl-induced seizures. Seizure threshold is depicted in µl of flurothyl necessary to induce specific seizure type (Mean±S.E.M.). (A) In females, tonic-clonic seizures in *Brd2+/-* mice had significantly lower threshold than in *Brd2+/+* littermate controls. (B) In males, clonic seizures in *Brd2+/−* mice had significantly lower threshold than in *Brd2+/+* littermate controls.

In females, the threshold for flurothyl-induced **tonic-clonic** seizures was statistically significantly (21%) lower in *Brd2* +/*−* (n = 12) compared to *Brd2* +/+ mice (n = 12; ANOVA F_(1,22)_ = 8.229; *p = 0.009; Figure 1A). The two covariates of age (range 3.5-8.0 months) and vaginal impedance were not found to be significant in any analysis and were removed from the analysis. (The lack of estrous cycle effect on flurothyl seizures is consistent with a previous report [38].) The threshold for **clonic seizures** in females did not differ between the genotypes (ANOVA F_(1,24)_ = 0.112; p>0.74), unlike male mice (see below). Some female mice developed multiple clonic seizures, but there was no difference between the *Brd2+/-* and *Brd2+/+* mice in the number of clonic seizures (Mann-Whitney U = 67.5; tied p>0.35). The threshold for twitches did not differ between *Brd2+/−* and *Brd2+/+* mice (ANOVA F_(1,24)_ = 0.076; p>0.78). The death rate was identical at 41.7% in *Brd2+/−* and *Brd2+/+* mice (Fisher's exact test p>0.99).

In *Brd2+/−* male mice (n = 16), the **clonic seizure** threshold was significantly decreased by 18% compared to the *Brd2+/+* males (n = 12; ANOVA F_(1,26)_ = 10.338; *p = 0.004; Figure 1B). Although some mice developed multiple clonic seizures, there was no difference in the total number of clonic seizures between the *Brd2+/+* and *Brd2+/−* mice (Mann-Whitney U = 77.5; tied p>0.24). There was no significant difference in the male mice (*Brd2+/−* vs. *Brd2+/+*) in the threshold for induction of twitches (ANOVA F_(1,20)_ = 1.901; p>0.18) or **tonic-clonic seizures** (ANOVA (F_(1,24)_ = 2.777; p>0.10). We always used one covariate (age: 3.5–8.0 months) on top of the main factor (genotype) but there was no effect of age on the threshold for flurothyl-induced twitches, clonic or tonic-clonic seizures, and no interaction of age with the main factor of genotype. Therefore, we removed age as a covariate in all statistics. Finally, the death rate did not differ between *Brd2+/−* (64.3%) and *Brd2+/+* mice (41.7%; Fisher's exact test p>0.43) male mice.

### Spontaneous seizures

**Figure 2:**
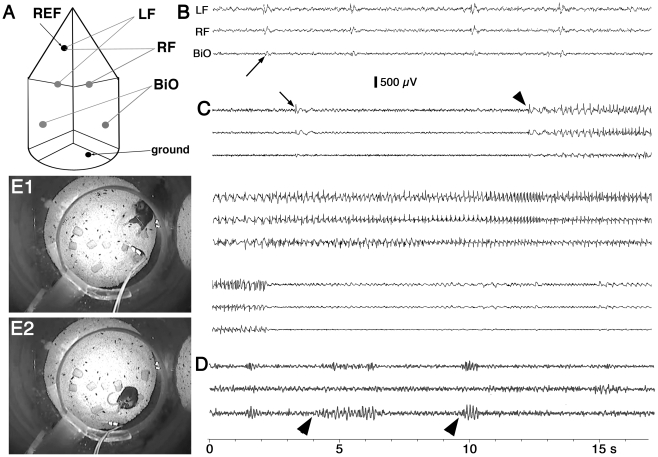
Combined EEG/videorecordings of spontaneous seizures in *Brd2+/−* mice. (A) Scheme of head mounted electrodes with one reference (REF) in the nasal bone, one common ground in the occipital area, and active electrodes in the left and right frontal area (LF, RF, respectively) and in both occipital areas (BiO). (B) EEG recordings of interictal discharges (one indicated by an arrow) in a *Brd2+/−* mouse associated with myoclonic jerks (twitches of body musculature). (C) EEG recordings from the same mouse showing a long EEG seizure consisting of spike-and-wave pattern. Onset of seizure is marked by an arrowhead. (D) EEG recordings of spindle-shaped sharp wave episodes associated with behavioral freezing in another *Brd2+/−* mouse. Onset of two spindles (about 3 s and 1 s long) is marked by arrowheads. (E) Frozen video frames (under infrared lighting) showing onset of a violent clonic seizure (E1) in a *Brd2+/−* mouse and the end of status epilepticus (after more than an hour of clonic seizures) in the same mouse (E2).

To determine whether *Brd2* haploinsufficiency is associated with spontaneous seizure development, *Brd2*+/*−* mice (females, n = 5) were subjected to long-term EEG/videomonitoring using two unipolar frontal and one bipolar occipital EEG channels and infrared video (Figure 2A). Three mice showed spontaneous seizure events, one of which showed interictal discharges (Figure 2B), sometimes associated with a whole body twitch. These interictal discharges developed into true spontaneous spike-and-wave clonic seizures lasting approximately 30 seconds each (Figure 2C). One *Brd2* +/*−* mouse had a total of three seizures of similar duration with an in-between interval of approximately 2 hours. A second *Brd2*+/*−* mouse had one brief clonic seizure that was recorded. The third mouse experienced relatively frequent episodes (0–19 per 24 hours) of behavioral arrest associated with EEG spindles of sharp waves, similar to human absence seizures (Figure 2D), and died in protracted clonic seizures (status epilepticus; Figure 2E) within 6 weeks of monitoring.

### Examination of GABAergic neurons

The decrease in the flurothyl-induced, primarily generalized seizure threshold and the presence of spontaneous primary generalized seizures in *Brd2*+/*−* mice suggested that there might be changes in the generalized seizure control system, which includes the SNR [32], and significant GABA involvement. We also examined other structures of this circuit [39]: primary motor cortex, caudate-putamen (CPu)/globus pallidus (GP), superior colliculus (SC), and ventral medial (VM) thalamic nucleus [40]. We always tested for sex differences, but in those analyses in which no male vs. female differences were detected, male and female data were combined.

**Figure 3:**
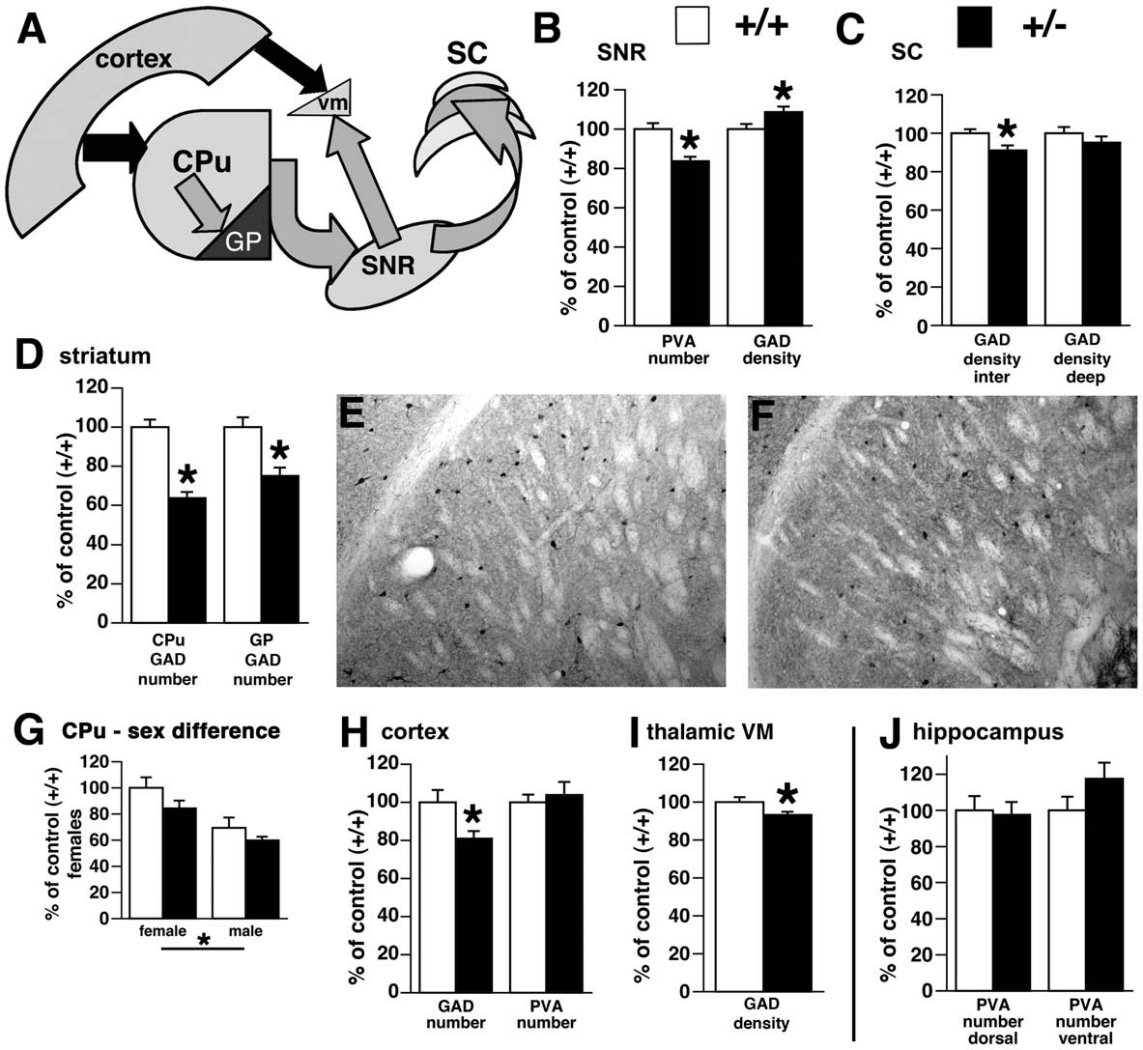
Expression of GABAergic markers in the *Brd2+/−* mice and *Brd2+/+ littermate* controls. (A) Simplified scheme of a direct basal ganglia pathway, which includes primary motor cortex, caudate-putamen (CPu) and globus pallidus (GP), substantia nigra reticulata (SNR), and its outputs the superior colicullus (SC) and ventral medial thalamic nucleus (VM). Dark arrows indicate glutamatergic outputs, while grey arrows mark GABAergic projections [39]. (B) Number of PVA-immunopositive neurons and density of GAD67 staining per neurons in the SNR of *Brd2+/−* mice (+/*−*) versus *Brd2+/+* littermate controls (*+/+*; expressed in % of control values; mean ±S.E.M.). While the number of PVA-positive neurons significantly decreased, the GAD67 content within neurons slightly increased in the SNR of *Brd2+/−* mice. (C) GAD67 content in the fibers of the intermediate gray matter layer (inter) of the SC was decreased in *Brd2+/−* mice vs. *Brd2+/+* controls. However, the GAD67 content in the deep gray matter layer of the SC (deep) was not different between *Brd2+/−* mice and. *Brd2+/+* controls. This finding is consistent with presence of GABAergic terminals originating from the SNR in the intermediate, but not deep, gray matter layer of the SC. (D) Number of GAD67-immunopositive neurons in the CPu and GP was profoundly decreased in both parts of the striatum in *Brd2+/−* mice compared to *Brd2+/+* littermates illustrated further in microphotographs of upper outer quadrant of CPu in a *Brd2+/+* (E) and *Brd2+/−* mouse (F). (G) Sex-specific difference in the relative number of PVA-positive neurons in the CPu. Males irrespective of genotype had about 30% fewer PVA neurons than females. There was no significant effect of genotype. (H) There was a significant decrease in number of GAD67-, but not PVA-immunopositive neurons in the primary motor cortex in *Brd2+/−* mice versus *Brd2+/+* controls. (I) GAD67 content in the fibers of the ventral medial thalamic nucleus (VM) in the *Brd2+/−* mice was significantly decreased compared to *Brd2+/+* controls. (J) Number of PVA-immunopositive neurons in the hilar area of the dentate gyrus in both dorsal and ventral hippocampi was unchanged as in *Brd2+/−* mice compared to *Brd2+/+* littermates.

In the SNR, parvalbumin (PVA) is almost completely co-expressed with GABA [41], thus serving as a marker of GABAergic neurons. Differences in the number of GABAergic neurons, which could directly affect seizure susceptibility [33], would thus be reflected in the numbers of PVA-expressing neurons. We found highly statistically significant differences in the numbers of GABAergic neurons in the SNR. *Brd2*+/*−* mice of either sex (n = 8; no sex differences present) had 20% fewer PVA-immunopositive cells in the SNR than did *Brd2*+/+ controls (n = 8; both sexes combined) (*p = 0.0008; Figure 3B).

We further hypothesized that the decreased number of PVA-immunopositive (i.e., GABAergic) neurons in the SNR of *Brd2+/−* mice could lead to a compensatory GABA overproduction correlated with an increased GAD67 (a GABA-synthesizing enzyme) content in those remaining GABAergic neurons. Thus, using densitometry [42], we examined the average GAD67 content in individual SNR cell bodies. There was a small (9%) but statistically significant increase (*p = 0.042; Figure 3B) in GAD67 expression in the SNR cell bodies in *Brd2*+/*−* mice (n = 8) vs. *Brd2*+/+ controls (n = 7), indicating a possible compensatory increase in GABA synthesis.

We next examined GAD67 concentration in fibers in the intermediate gray matter layer of the SC. This structure contains SNR output and also plays a role in generalized seizure control [43]. It is important to note that the GAD67 concentration in fibers in the SNR target areas (such as SC or VM) is the product of the number of GAD67-positive SNR cells and their GAD67 content. We measured areas devoid of cell bodies to look for differences in the levels of GAD67 in axons emerging from the SNR GABAergic neurons. We found a small (10%) but statistically significant decrease in GAD67 immunodensity in *Brd2+/−* mice (n = 7) compared to *Brd2+/+* mice (n = 8; *p = 0.018; Figure 3C) that was also not sex-related. For an internal control, we examined the deep gray matter layer of the SC (with negligible SNR inputs) and found no difference in *Brd2+/−* vs. *Brd2+/+* GAD67 immunodensity (Figure 3C). The data suggested that the changes in the numbers of GABAergic neurons (PVA-positive) and in the levels of GAD67 within those GABAergic cells that are found are pathway-specific, possibly limited to basal ganglia connections.

In the striatum, we determined relative numbers of both GAD-67- and PVA-immunopositive neurons. In both parts of the striatum, the caudate/putamen (CPu) and globus pallidus (GP), we found highly statistically significant decreases in numbers of GAD67-immunopositive neurons in *Brd2*+/*−* mice (n = 7). In the CPu, there was a 67% decrease compared to *Brd2+/+* mice (n = 7; p<0.0001; Figure 3D). In the GP, the decrease in GAD67-immunopositive neurons in *Brd2+/−* mice was 32% (*p = 0.0026; Figures 3D, E, F). Again, there were no sex-related differences.

Although we noted above that there was a major effect of genotype on GAD67-immunopositive neurons, we saw no effect of *Brd2* genotype on PVA-immunopositive neuron numbers in either CPu or GP, suggesting a structure-specific expression of GABAergic neurons in *Brd2+/−* mice. However, two-way ANOVA revealed a significant effect of sex alone, consistent with previously reported prenatal findings [44]: males had, on average, 30% fewer PVA-immunopositive neurons compared to females irrespective of genotype (*p = 0.0017; Figure 3G). This was unlike the findings in the SNR, in which there was a notable genotype-dependent difference in PVA-positive neuron count.

We also compared the number of GAD67- and PVA-immunopositive neurons in the primary motor cortex. As in the CPu and GP, there was a statistically significant 23% decrease in the number of GAD67-positive neurons in *Brd2+/−* mice (n = 7) vs. *Brd2+/+* mice (n = 6; *p = 0.025; Figure 3H). However, like the CPu and GP and unlike the SNR, there was no difference in the number of PVA-immunopositive neurons in the motor cortex. Unlike the CPu and GP results (above), there was no effect of sex on the numbers of either neuronal subtype (Figure 3H).

Finally, we determined GAD67 concentration in areas of fibers devoid of cell bodies in the thalamic VM, another significant GABAergic output of the SNR, involved in seizure control [40]. Similar to SC, we found a small (7%) but statistically significant decrease in GAD67 immunodensity in *Brd2+/−* mice (n = 8) compared to *Brd2+/+* mice (n = 8; *p = 0.048; Figure 3I). There was no effect of sex on the GAD67 concentration and no interaction between the sex of the subjects and genotype.

Because the hippocampus is not involved in primarily generalized seizures [45], we hypothesized that there will be no differences in GABA markers in this structure. The distribution of GABA markers in the hippocampus could thus indicate whether the GABA-related changes in *Brd2+/−* mice represent a global brain feature or are specific for the basal ganglia pathway. There were no genotypic or sex-related differences in PVA-immunopositive neuron numbers between *Brd2+/−* and *Brd2+/+* mice in the dentate gyrus in the ventral and dorsal parts of the hippocampus, suggesting the effect of a Brd2 deficit was confined to only certain pathways (Figure 3J).

## Discussion

This is the first demonstration of a developmentally-related mechanism for seizure susceptibility of common forms of epilepsy. That mechanism involves a deficit of GABAergic neurons caused by haplo-insufficiency of the mouse *Brd2* gene. This significant deficit of inhibitory GABAergic neurons was observed along the basal ganglia seizure-controlling pathway, but not in regions of the brain outside this pathway (i.e., deep layers of the superior colliculus not connected to the SNR or hippocampal dentate gyrus). This decrease in inhibitory neurons and their GABA-synthesizing enzyme expression (GAD67) presages increased seizure susceptibility and spontaneous seizure development.

Even though the effects of a *BRD2* deficiency in humans are likely to be more subtle than those arising from a complete elimination of one of the *BRD2* alleles (the situation in the *Brd2*+/*−* mice), there are consistent imaging-related differences in JME patients vs. normals [46], [47], [48], [49]. That is, there are additional human findings, besides the genetic data, that directly connects human epilepsy to our finding of a reduction in GABAergic neurons in *Brd2*+/*−* mouse basal ganglia pathway. Decreased volume in the basal ganglia was reported in human IGE [50], [51] and a decrease in caudate nucleus blood-oxygen-level-dependent (BOLD) MRI signal has been observed in children with absence epilepsy [52]. Also, after treatment of IGE patients with lamotrigine, there is reduced glucose metabolism in basal ganglia (including the substantia nigra), and in cerebral cortex and thalamus, further suggesting association of basal ganglia pathway with IGE syndromes in humans [53]. These findings correlate well with our data in the *Brd2+/−* mouse model.

Our data show that haploinsufficiency for the *Brd2* gene in mice is associated with decreased number of GABAergic neurons, which may be important for control of seizure activity, especially in critical brain structures [54], [55], [56]. These results strengthen the hypothesis that aberrant expression of human *BRD2* contributes to JME susceptibility, and perhaps other epilepsy-related phenomena, in humans. That is, GABAergic system impairment in *Brd2*+/*−* mice suggests that a developmentally-based deficit of GABAergic neurons might be a mechanism underlying the etiology of IGE. In this study, we focused our observations on the GABA system because we found increased susceptibility to primarily generalized seizures and generalized spontaneous seizures. In these seizure syndromes, structures and pathways containing GABA are considered as major contributors [33], [56], [57], [58], [59]. However, we cannot exclude participation of enhanced activity of glutamatergic excitatory system in our findings. Further, the sex-related differences we observed in the mice with a Brd2 deficit correlate well with the sex-specific expression of seizure susceptibility in human JME [60].

Thus, regulatory genes such as the bromodomain-containing *BRD2* may be significantly involved in common IGE syndromes. The recent report of a role for the gene encoding the transcription factor ELP4 in the development of centrotemporal EEG spikes in rolandic epilepsy [61] further suggests that the common genetic epilepsies can be influenced by genes involved in the regulation of gene expression in brain development. The basis for autosomal dominant partial epilepsy with auditory features (ADPEAF), the LGI1 gene, also appears to have a developmental origin [62]. These findings are important for widening our perspectives on which kinds of genes are responsible for IGE syndromes in particular and seizure disorders in general.

An additional contribution of this study is the focus on somewhat neglected subcortical basal ganglia structures [36] specifically with regard to IGE. This focus represents a diversion from the common approach of concentrating on the hippocampus, amygdala, thalamus, or cortex, with regard to seizure disorders, and indicates that specific seizure disorders may involve specific brain structures. This finding may significantly contribute to novel therapies focused on those brain nuclei in patients with IGE.

In conclusion our data clearly indicate that *Brd2* haploinsufficiency is associated with a deficit of GABAergic neurons and along the basal ganglia path in structures critical for control of seizure activity. This developmentally-related impairment of the GABAergic system likely contributes to increased susceptibility to provoked seizures and to the development of spontaneous seizures in *Brd2+/−* mice. *BRD2* has been associated and linked to the expression of JME in humans, this underlying susceptibility could, when another insult occurs, either environmental or genetic, lead to the expression of epilepsy.

## Materials and Methods

### Ethics Statement

All animal utilization was carried out in strict accordance with the recommendations in the Guide for the Care and Use of Laboratory Animals of the National Institutes of Health. The protocols were approved by the IACUC of the Columbia University (breeding; protocol number: AAAA8870) and the Albert Einstein College of Medicine (experiments; protocol number: 20080914). All surgery was performed under ketamine/xylazine anesthesia, and all efforts were made to minimize suffering and the number of mice used while keeping the statistical power.

*Brd2+/−* mice (*Mus musculus*) and *Brd2+/+* littermates (controls) were generated by mating heterozygous *Brd2+/−* females to heterozygous *Brd2+/−* males. The mice used in this study were at the 7^th^ generation of backcrossing onto a C57Bl/6J background. At this step of backcrossing, their genetic background would be considered on average 99% C57Bl/6J (Mouse Nomenclature, jaxmice.jax.org). The genotypes of the mice were assessed by PCR using primers that spanned the gene-trap vector junction inserted into the *Brd2* gene [20].

Vapor inhalation of flurothyl [bis(2,2,2-trifluorethyl) ether; CAS 333-36-8], induces a sequence of seizure behaviors: myoclonic twitches, clonic seizures of face and forelimb muscles with preserved righting ability, and tonic-clonic seizures of all four limbs with the loss of righting [63]. Mice in pairs (always *Brd2+/−* and *Brd2+/+*) were exposed to flurothyl in an air-tight chamber. Flurothyl was delivered at a constant 40 µl/min rate until the tonic-clonic seizures developed. Seizure behavior was evaluated by two independent observers blinded to the mouse genotype. Latency to onset of twitches, clonic, and tonic-clonic seizures were recorded. The amount of flurothyl delivered by the time of seizure onset served as the measure of the seizure threshold value [64]. Female estrous cycles were monitored as changes in vaginal epithelium impedance using a vaginal impedance meter (Fine Science Tools, Foster City, CA) [65], [66], [67] because changes in progesterone and estrogen levels may alter seizure susceptibility [68].

Pinnacle Technology, Inc., three-channel EEG system with time-locked infrared videorecording was used for long-term EEG/videomonitoring. Mice were implanted with EEG electrodes under ketamine/xylazine (70/7 mg/kg i.p.) anesthesia. We used epidural silver ball electrodes, positioned bilaterally in frontal and occipital cortex. A stainless steel screw in the nasal bone was the reference electrode, and a similar screw above the cerebellum served as ground. The mice were recorded for minimum of 5 days, 24 hours a day. The recordings were first pre-sorted using Sirenia Seizure software and all seizure-suspect segments were visually inspected including video.

To identify GABAergic neurons, 40 µm thick coronal hemi-sections, were cut. Alternating sections were collected for parvalbumin (PVA) and GAD67 immunostaining. PVA is a useful marker of GABAergic neurons in the SNR or hippocampal hilus, where it almost completely co-expresses with GABA [41]. However, in other brain structures PVA is expressed only in small subpopulations of GABAergic neurons. GAD67 (isozyme of glutamic acid decarboxylase, a GABA synthesizing enzyme) tags almost entire population of GABAergic neurons and additionally, GAD67's expression (density measurement) within individual cells may provide an estimate of GABA synthesis. The immunostaining was performed in free-floating sections using an avidin-biotin horseradish peroxidase method (Vectastain AB kit, Vector Laboratories, Burlingame, CA) [69] with primary antibodies: anti-parvalbumin (1∶5000; Sigma, St. Louis, MO); anti-GAD67 (1∶4000; Millipore, Temecula, CA). We were interested only in differences in cell numbers between *Brd2+/−* and *Brd2+/+* mice; therefore, we compared the relative number of immunopositive cells in *Brd2+/−* and *Brd2+/+* mice. Section images were digitally captured. Counts were performed in the direct basal gaglia pathway (primary motor cortex, striatum, SNR) and in the hilus of the dorsal and ventral hippocampus to determine site-specificity of findings. For counting, a minimum of three corresponding sections were selected in each structure of interest in both *Brd2+/−* and *Brd2+/+* mice and all immunopositive neurons were counted. Counts were averaged and the average used for statistical evaluation.

We also compared relative densities of GAD67 expression in *Brd2+/−* and *Brd2+/+* mice. To minimize variability between the groups, tissue from a heterozygote and a wild type mouse were processed together. We evaluated the density of GAD67 immunoexpression in the SNR cells (manually outlined somata) and in the fibers of the superior colliculus (SC) or ventral medial thalamic nucleus (VM) [40], [42] under 400× magnification using computer-assisted image analysis (ImageJ, Wayne Rasband, NIH) to quantify the relative amounts of protein [70]. In three corresponding sections per subject, we averaged semiquantitative densitometry measurements of three samples of areas (same area rectangles randomly positioned over the section) containing fibers or five randomly chosen immunopositive cell somata. In the SC, we measured GAD67 expression in the intermediate (receiving abundant SNR input connections) and deep (sparse SNR inputs) gray matter layers [71]. Three areas devoid of cell bodies were manually outlined across each layer. All densitometry measurements were normalized using in relation to white matter background (non-specific staining in the cerebral peduncle for the SC or mamillothalamic tract for the VM) and averaged for each subject before entering statistics.

#### Statistics

Seizure susceptibility was evaluated by sex. For seizure threshold evaluation, multivariate ANOVA was used. Genotype was the main factor (levels: *Brd2+/+* or *Brd2+/−*). Age was used as a covariate in males; in females, age and vaginal impedance were the covariates. A lack of effect of a covariate, and no interaction with the main effect, caused the covariate's removal from the analysis. The numbers of clonic seizures were compared by non-parametric Mann-Whitney U test. Immunopositive cell numbers were compared first using ANOVA with factors of genotype and sex. If no main effect of sex was found, the factor of sex was removed from analysis and the evaluation was run using Student's t-test. Densitometric expression of GAD67 was evaluated similarly. Significance threshold was set at p<0.05 and adjusted for multiple comparisons. Graphs show means ±S.E.M.s.
